# “They do not see us as one of them”: a qualitative exploration of mentor mothers’ working relationships with healthcare workers in rural North-Central Nigeria

**DOI:** 10.1186/s12960-018-0313-9

**Published:** 2018-09-10

**Authors:** Nadia A. Sam-Agudu, Angela Odiachi, Miriam J. Bathnna, Chinazom N. Ekwueme, Gift Nwanne, Emilia N. Iwu, Llewellyn J. Cornelius

**Affiliations:** 1 International Research Center of Excellence, Institute of Human Virology Nigeria, Plot 252 Herbert McCaulay Way, Abuja, Nigeria; 2 Division of Epidemiology and Prevention, Institute of Human Virology, University of Maryland School of Medicine, 725 West Lombard Street, Baltimore, MD 21201 United States of America; 3 Abuja, Nigeria; 4 School of Nursing, Rutgers University, 180 University Avenue, Newark, NJ 07102 United States of America; 5 Care and Support Program, Institute of Human Virology Nigeria, Plot 252 Herbert McCaulay Way, Abuja, Nigeria; 6 School of Social Work and College of Public Health, University of Georgia Athens, 279 Williams St, Athens, GA 30602 United States of America; 7 Department of Community Medicine, University of Nigeria College of Medicine, Enugu, Nigeria

**Keywords:** Mentor mother, Expert mother, Peer support, PMTCT, HIV, Task shifting, Human resources for health, Nigeria

## Abstract

**Background:**

In HIV programs, mentor mothers (MMs) are women living with HIV who provide peer support for other women to navigate HIV care, especially in the prevention of mother-to-child transmission of HIV (PMTCT). Nigeria has significant PMTCT program gaps, and in this resource-constrained setting, lay health workers such as MMs serve as task shifting resources for formal healthcare workers and facility-community liaisons for their clients. However, challenging work conditions including tenuous working relationships with healthcare workers can reduce MMs’ impact on PMTCT outcomes. This study explores the experiences and opinions of MMs with respect to their work conditions and relationships with healthcare workers.

**Methods:**

This study was nested in the prospective two-arm *Mo*ther *Ment*or (MoMent) study, which evaluated structured peer support in PMTCT. Thirty-six out of the 38 MMs who were ever engaged in the MoMent study were interviewed in seven focus group discussions, which focused on MM workload and stipends, scope of work, and relationships with healthcare workers. English and English-translated Hausa-language transcripts were manually analyzed by theme and content in a grounded theory approach.

**Results:**

Both intervention and control-arm MMs reported positive and negative relationships with healthcare workers, modulated by individual healthcare worker and structural factors. Issues with facility-level scope of work, workplace hierarchy, exclusivism and stigma/discrimination from healthcare workers were discussed. MMs identified clarification, formalization, and health system integration of their roles and services as potential mitigations to tenuous relationships with healthcare workers and challenging working conditions.

**Conclusions:**

MMs function in multiple roles, as task shifting resources, lay community health workers, and peer counselors. MMs need a more formalized, well-defined niche that is fully integrated into the health system and is responsive to their needs. Additionally, the definition and formalization of MM roles have to take healthcare worker orientation, sensitization, and acceptability into consideration.

**Trial registration:**

Clinicaltrials.gov number NCT01936753, registered September 3, 2013.

## Background

In the context of HIV service delivery, mentor mothers (MMs), also known as expert mothers, are HIV-positive women with first-hand experience as exemplary clients in the prevention of mother-to-child transmission of HIV (PMTCT) who provide experiential guidance for other women living with HIV [1–5]. Beyond their personal experience, MMs often receive training to enhance their peer support services and work alongside formal healthcare workers (HCWs) [1, 4, 5]. In this regard, MMs can be considered lay health workers: lay people who have been trained for short periods to assist formal HCWs and take over certain tasks [6–9]. MMs and similar lay HIV health workers often do not have specific qualifications other than being persons living with HIV [4, 10–12]. They work in health facilities, in clients’ homes, and in the larger community and ultimately act as a link between health facilities and communities [4, 6, 10, 11].

Depending on the setting, MM roles at health facilities include HIV testing, pre- and post-test counseling, enrolling clients into PMTCT/HIV care, booking client appointments, assisting in drug dispensing and adherence counseling, and tracking clients who have missed appointments or dropped out of care [12–16]. These MM roles are played largely in the framework of task shifting, which the World Health Organization (WHO) defines as “the rational redistribution of tasks among health workforce teams” [17]. In HIV care, task shifting may occur from doctors to nurses or other formal HCW cadres [18, 19] and from doctors, nurses, and other formal HCW cadres to lay health workers [4, 11, 19, 20]. The professional-to-layperson model of task shifting has been formally or informally adopted to various degrees in several sub-Saharan African countries to facilitate scale-up of HIV (including PMTCT) services, reach and retain clients, reduce disease burden, and ultimately improve treatment and prevention outcomes [4, 10, 11, 19, 20]. Task shifting is particularly helpful in low-resource settings where there is a shortage of human resources for health with concomitant high burden of disease.

At 3.2 million, Nigeria has the second largest population of people living with HIV globally, after South Africa [21]. Furthermore, Nigeria has a large PMTCT burden, along with wide program gaps: only 30% of approximately 200 000 HIV-positive and pregnant Nigerian women receive antiretroviral drugs annually, and only 9% of HIV-exposed infants receive timely early infant diagnosis testing [22, 23]. In Nigeria, structured MM peer support has been shown to improve maternal retention and viral suppression [24] as well as timely infant presentation for HIV testing [25]. Similar findings on the positive impact of peer support on PMTCT outcomes have been reported from other African countries [1, 26–30].

Despite the well-established benefits of peer support in PMTCT, many high-burden countries—including Nigeria—have not formally adopted these interventions at the national level. For example, in 2005, Nigeria introduced expanded roles for lay voluntary workers including people living with HIV as expert patients in pilot projects such as the Integrated Management of Adult and Adolescent Illness [31]; this was however not fully implemented nationwide. Both the 2009 national decentralization program for HIV treatment scale-up to primary healthcare centers and the 2014 national task shifting/task sharing guidelines [32] formalized policies to only support expanded roles of non-physician health workers already in the Nigerian civil service structure (e.g. nurses, midwives, community health officers, community health extension workers and pharmacy attendants). MMs and other HIV-positive treatment supporters are not part of the current civil service structure and are typically supported by externally funded implementing partners.

Furthermore, challenges exist in terms of defining MMs’ roles/niche in PMTCT/HIV programs in particular and the formal health system in general [7, 9, 13, 14, 33]. Under these circumstances, professional relationships between MMs (as HIV-positive lay health workers) and the formal HCWs who supervise them may be complicated. Placed in this hierarchical environment, MMs, with often poorly defined roles, little or no education or professional credentials, low wage-earning capacity, and known to be HIV-positive, may be highly vulnerable to stigma, discrimination, marginalization, non-supportive supervision, or other negative experiences at the healthcare facility [10, 12–14, 34].

The nature of MM’s working conditions, especially HCW-MM working relationships, have so far not been well-characterized in Nigeria, a country which stands to gain significantly from the scale-up of peer support interventions in its challenging PMTCT program. This paper explores the nature of the working environment for MMs at primary healthcare centers in rural North-Central Nigeria. Specifically, we aim to describe, from the perspective of MMs, how interactions with healthcare workers shape MMs’ working conditions and influence their performance.

## Methods

### Study design

This qualitative study was nested within a larger PMTCT implementation research project, the MoMent (*Mo*ther *Ment*or) study, in North-Central Nigeria. MoMent was a prospective cohort study that compared a standardized, closely supervised MM program with the less-structured, less-supervised routine MM program at primary healthcare centers in rural North-Central Nigeria [35]. Main outcomes included postpartum maternal retention and viral suppression and timely uptake of early infant diagnosis [35]. This article draws its findings from focus group discussions (FGDs) conducted towards the end of the prospective study follow-up, to capture the experiences and opinions of all MoMent MMs (intervention and control) regarding their roles and working conditions during study implementation.

### Study setting and population

This study was conducted in rural communities of the Federal Capital Territory and Nasarawa State in North-Central Nigeria. Study participants were MMs engaged at all 20 (10 intervention and 10 control) primary healthcare centers that served as MoMent study sites. Table 1 compares training, supervision and scope of work for all MoMent MMs working in both intervention and control arms [24, 35]. MoMent MMs in both arms were chosen from communities surrounding the primary healthcare centers they were assigned to. All of these women had completed the PMTCT cascade at least once and were expected to guide other women in navigating and being compliant with PMTCT services. MMs in both arms were expected to work at both facility and community-level and were provided the same stipend amount. The major differences between the two MM groups were in the supervision and structure built into the intervention arm: intervention MMs received baseline training via a standard curriculum with daily, hands-on supportive supervision from a study-designated MM supervisor. Furthermore, all intervention MMs utilized standardized logbooks for documenting client calls and visits. Random quarterly performance audits were conducted via client feedback, in order to improve and/or maintain MM work performance in the intervention arm [36].

**Table 1:** MoMent peer support program description, roles, and responsibilities

	Control arm mentor mother	Intervention arm mentor mother
Program description and requirements		
HIV-positive woman	Yes	Yes
Experiential PMTCT knowledge	Yes	Yes
Standard pre-engagement selection criteria^a^	No	Yes
Standard baseline training and curriculum with certification^b^	No	Yes
Stipend for work activities	Yes (~ 50 USD monthly)	Yes (~ 50 USD monthly)
Schedule for client interaction^c^	No	Yes
Scope of client-related work formally communicated^d^	No	Yes
Facility or community-based activities	Both	Both
Linked to HIV+ clients at ANC clinic	Yes	Yes
Activity documentation	Weak, not standard, not consistently linked to PMTCT outcomes, often not reviewed	Emphasized, standard logbook provided, PMTCT outcomes-specific, reviewed by MM supervisor
Supportive supervision	Weak support and supervision, responsibility of facility staff-in-charge	Emphasized, responsibility of supportive MM supervisor who reports to staff-in-charge
Audits of client interaction activities^e^	No	Yes
Formal performance evaluations	No	Yes, based on client feedback
Re-orientation or disengagement based on performance	No	Yes
Peer support activities		
Document client interactions	Yes, inconsistent (in non-standardized notebooks)	Yes, required (in standardized, outcomes-specific logbook)
Conduct HIV tests for ANC clinic clients	No	No
Immediately flag clients with missed appointments	No	Yes
Track clients with missed appointments	Yes, after several weeks	Yes, required within 3 days
Daily feedback sessions with supervisor	No	Yes

*PMTCT* prevention of mother-to-child transmission of HIV, *USD* US dollars, *ANC* antenatal care, *MM* mentor mother

^a^HIV-positive, 18–45 years old, successfully completed PMTCT program at least once; speaks ≥ 1 local language; disclosed to partner/family member; willing to disclose HIV status to peers

^b^5-day, 17-module training including pre- and post-test and counseling role-play

^c^Home visit within 5 days of client assignment, twice monthly visits, visit within 3 or 7 days if non-facility or facility delivery, respectively

^d^Via written contract provided and explained to all MMs in English or other language they understood

^e^One randomly selected client interviewed every 6 months to validate MM reports on interactions with client and for client feedback on MM performance

### Participant recruitment

Over its 5-year implementation period, MoMent engaged a total of 38 MMs across both intervention (structured peer support) and control (unstructured peer support) sites. The number of MMs assigned per site was guided by a ratio of 1:10–15 between MMs and pregnant or postpartum clients (up to 18–24 months post-delivery) [35]. Ultimately, intervention arm MMs had an average ratio of 1:12 while control MMs averaged 1:14 clients [24].

All MoMent ever-engaged MMs were eligible to participate in the MM FGDs. Towards the end of the study, all 38 MMs (regardless of whether still actively engaged or not) were contacted by telephone, by research officers who had been stationed at each MoMent site during the study. MMs were not contacted or recruited by healthcare workers for these FGDs. The research officers briefed each of the 38 MMs about the FGDs; possible dates and the MMs’ availability on those dates were discussed. A total of 36 out of the 38 MMs indicated their interest and availability to participate in the FGDs. These 36 interested and available MoMent MMs were provided information on the date and location of their specific FGD. Two MMs, both from the control arm, were interested; however, they were unable to participate in the FGDs on the scheduled dates—one was recovering from surgery while the other had to travel out of town.

All 36 successfully recruited MMs presented for the FGDs, which were conducted on non-clinic days in private rooms at study primary healthcare centers within the communities where the MMs worked, or at a private venue within their work catchment area. This was done to preserve confidentiality and to encourage discussions on this topic without fear of victimization by facility healthcare workers. Written informed consent was obtained from all MMs before the FGDs. Snacks and transport reimbursement (depending on distance traveled) were provided on the day of the FGD for all participants. Healthcare workers neither participated in nor observed the FGDs.

### Data collection

Seven FGDs (four among intervention MMs and three with control MMs) were conducted between September and November 2016, which marked the end of the 6-month postpartum follow-up for all participants for the prospective study’s primary outcomes. Each of the seven FGDs conducted had four to six MM participants, ultimately representing all 20 MoMent sites.

Prior to each FGD, an interviewer-administered form was used to capture participants’ socio-demographic information including educational attainment, marital status, religious affiliation, parity and duration of engagement as MMs.

Each discussion was audio-recorded and guided by a trained bilingual (English and Hausa) facilitator, with or without a co-facilitator, and an observer. During each FGD, at least one observer took notes on non-verbal cues, which were used to assist in data analysis and interpretation. All facilitators and observers were study staff familiar to participants, due to their interactions with these MMs at study sites during MoMent data collection. All study FGD staff had a minimum of 2 years working experience with the MoMent study and had had at least 1 year experience in conducting qualitative interviews. None of the FGD facilitators or observers worked as facility staff nor had any supervisory role over MMs participating in their respective FGDs.

The FGD guide explored MMs’ opinions on their workload and stipends, terms of engagement, scope of work, and relationships with healthcare workers. Each FGD lasted for 60–90 min.

Since the number of FGDs to be conducted was limited by the specific number of MoMent MMs engaged and available, data saturation was not a consideration. However, after transcription and analysis of the initial seven FGDs, we conducted a “member check” to gain participant feedback on the initial findings and to validate the collected data and its interpretation [37]. A cross-section (*n* = 4) of the 36 original participants were recruited for the member check; these participants were selected in order to equitably represent religion, high and low levels of education, and intervention and control arms. The member check was performed in September 2017 as a group discussion, where the key findings from qualitative data analysis were presented to participants for confirmation, correction, and additional commentary. Ultimately, the member check was in agreement with initial findings. The member check was conducted by the same facilitators and observers who implemented the initial FGDs.

### Data transcription and analysis

Audio-recorded FGDs were transcribed verbatim in English or transcribed and translated from Hausa to English were necessary. Manual transcription and analysis were performed by the same facilitators and observers who conducted the FGDs. For data analysis, we adopted the constant comparative method in a grounded theory approach [38]. In this approach, inductive methodology is used to systematically generate theory from the data collected. We selected a series of code words to develop themes and sub-themes from the qualitative data. Preset codes were related to the general themes in our FGD guides and served as the root of our coding tree. The root pre-set code words for our coding tree were “work relationships,” “stigma/discrimination,” “working conditions and pay,” and “roles/responsibilities.” Eight paired analysts independently coded and analyzed the data. This was followed by group review, triangulation, and content analysis by iteration until a final consensus on patterns and categorizations was achieved. The research consultant (AO) additionally independently analyzed and coded data with Nvivo 11 (QSR International, Victoria, Australia) and compared the findings to emerging themes identified by the paired researchers.

## Results

Of the 36 MMs interviewed, 32 (89%) had worked with the MoMent study for at least 2 years; the remaining four (11%) had worked with MoMent for at least 1 year. Twenty-five of the 36 (69.4%) participants had worked as MMs for between 2 and 5 years (Table 2); 80.6% (29/36) of these women had all living children confirmed HIV-negative. Table 2 presents details of participants’ socio-demographics. Median age of MMs was 32 years, and 18 (50%) were married; notably, 14 of the 18 single women (77.8%) were widowed.

**Table 2:** Sociodemographic profile of mentor mothers interviewed

Characteristics	Control mentor mothers (*N* = 15) *n* (%)	Intervention mentor mothers (*N* = 21) *n* (%)	All mentor mothers (*N* = 36) *n* (%)
Age, years*	32.0 (28.5–35.0)	32.5 (28.8–37.3)	32.0 (28.5–36.0)
No response	0	1	1
Educational attainment			
None	0 (0.0)	0 (0.0)	0 (0.0)
Qu’ranic only	2 (13.3)	0 (0.0)	2 (5.6)
Primary	4 (26.7)	6 (28.6)	10 (27.8)
Secondary	4 (26.7)	13 (61.9)	17 (47.2)
> Secondary	5 (33.3)	2 (9.5)	7 (19.4)
Religious affiliation			
Christian	8 (53.3)	18 (85.7)	26 (72.2)
Muslim	7 (46.7)	3 (14.3)	10 (27.8)
Marital status			
Single	1 (6.7)	2 (9.5)	3 (8.3)
Married	4 (26.7)	13 (61.9)	17 (47.2)
Divorced	0 (0.0)	1 (4.8)	1 (2.8)
Widowed	10 (66.7)	5 (23.8)	15 (41.7)
No. of living children			
None	1 (6.7)	0 (0.0)	1 (2.8)
1–2	7 (46.7)	6 (28.6)	13 (36.1)
3–4	5 (33.3)	13 (61.9)	18 (50.0)
≥ 5	2 (13.3)	2 (9.5)	4 (11.1)
No. of years working as mentor mother			
1	0 (0.0)	2 (9.5)	2 (5.6)
2–3	6 (40.0)	6 (28.6)	12 (33.3)
4–5	6 (40.0)	7 (33.3)	13 (36.1)
≥ 6	3 (20.0)	6 (28.6)	9 (25.0)
Other income generation activity besides peer counseling?			
Yes	13 (86.7)	15 (71.4)	28 (77.8)
No	2 (13.3)	6 (28.6)	8 (22.2)

*Displayed as median (interquartile range)

### Findings from focus group discussions

**Fig. 1:**
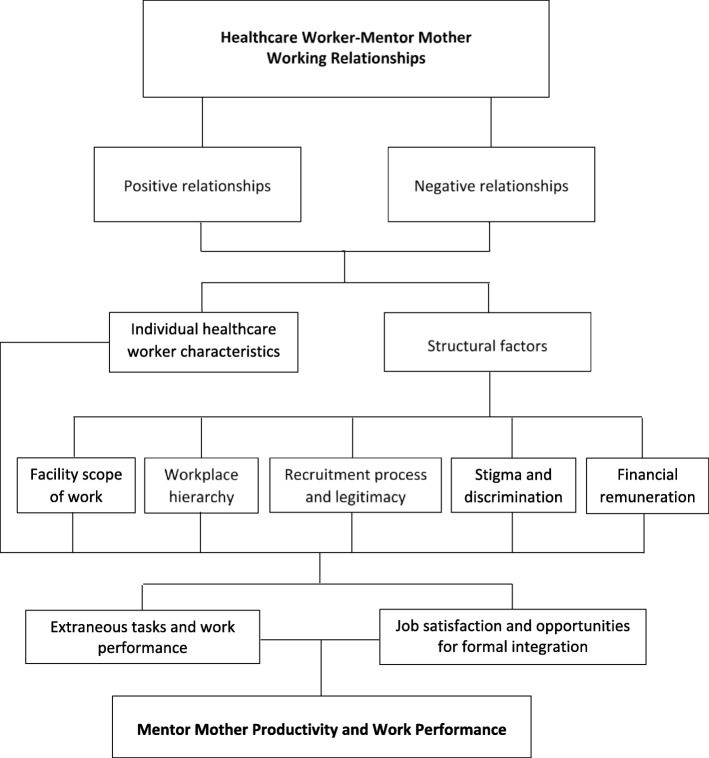
Core emerging themes from qualitative data analysis

Figure 1 displays the core themes that emerged from data analysis and interrelationships identified.

#### Work relationships between healthcare workers and mentor mothers

MMs, much like other lay health workers, are typically accountable to, and supervised by clinical staff, often nurses. Thus, their work conditions—roles, work hours, routines, and integration or marginalization in the clinic—are significantly controlled by these professional health workers. In this study, we noted a mixture of both positive and negative healthcare worker-mentor mother working relationships. For example, MMs reported relationships with some HCWs as cordial, where the latter were supportive of MMs and their work in the clinic.

The facility staff helps us with the drug appointment dates of the clients and he enters the client’s information in our note books.—Control MM FGD3

My facility staff helps me with some of my duties and whenever I return, they update me on all that had happened while I am away. They call me to check up on me and encourage me to show up for work.—Intervention MM FGD4

Everyone at my facility is very supportive.—Intervention MM FGD1

Despite these examples of cordial relations, some MMs felt alienated and not appreciated as fellow health workers by the HCWs they worked with, highlighting the relative lack of MMs’ integration into their work environment.

They treat us like we are not part of them; we are not among the staff. Whenever they have meetings, they don’t let us join. They don’t see us as one of them. They involve others and exclude us. The mentor mothers are nothing.—Intervention MM FGD3

On ANC days, we work together, do everything together. But if there is anything [of benefit], they will say, leave, are you one of the staff? …when it is time to share they will say it’s for the staff… when they see a positive mother it is then that they remember us.—Intervention MM FGD3

MMs also discussed how some HCWs would denigrate them and their work performance:

They make comments questioning our ability to carry on our duties even to the point of threatening to report us.—Control MM FGD1

#### Structural and individual healthcare worker characteristics influencing work relationships

Mentor mother reports suggest that individual HCW characteristics and structural factors influenced the nature of HCW-MM relationships.

The last matron we had in Facility A, she gave me a “heart attack”. As soon as I get to the gate of the clinic my heart always skips because I know it will be trouble all through…But with this new one, I do not have any problems.—Intervention MM FGD3

Structural issues emerging from the discussions included poorly defined and/or communicated MM scope of work, MMs’ recruitment process, and their legitimacy and hierarchy within the health facility.

#### Poorly defined scope of work at the facility

Under MoMent’s structured peer support program, MMs had a clearly specified scope of work, which was to be devoted largely to client interaction at the facility and/or in the community (Table 1) [35]. Peer support in the control arm was less well-defined and did not use standardized tools. However, similar to the control arm, intervention MMs’ scope of work at the facility was not well-defined, especially vis-à-vis HCWs’ roles. FGDs highlighted the fact that many MMs were confused about their roles at the facility:

I don’t know exactly what registers we are supposed to handle and those we should not be responsible for, because we get conflicting information … This clarification will make me focus on those registers that are my responsibility and reject any unrelated tasks.—Intervention MM FGD1

Some MMs also noted that they were performing tasks that they regarded as clearly outside their scope of work.

Sometimes, they [HCWs] make us do jobs that are not part of our responsibilities as peer counselors... They give us additional jobs apart from the peer counseling job.—Control MM FGD3

I handle the ANC register. If you do not do that, the nurses will begin to embarrass you. The in-charge will say things like ‘you don’t want to assist us in the facility’.—Intervention MM FGD1

Sometimes we dispense drugs*.*—Control MM FGD1

They show us how to refill drugs for ANC clients and we assist them in doing that.—Control MM FGD2

The extraneous tasks MMs performed also extended to non-clinical duties, some of which they performed willingly or volunteered for:

We mop, we wash the toilets, but we are just helping out at the facility.—Control MM FGD1

They do not ask us. We have volunteered ourselves to help when we are less busy with our peer counselling responsibilities.—Control MM FGD2

Any extra thing I do in the facility will be on a volunteer basis like buying of food for the in-charge which is something I do willingly, not because it’s part of my job.*—*Intervention MM FGD1

Despite the willingness to assist, MMs considered some of these tasks unacceptable.

Due to insufficient staff strength, I assist in the card room, I file folders and it is not my job. Yet they still tell me to sweep and mop! I agree I will bring out cards and file documents but the sweeping and the mopping, I do not want to do it.*—*Control MM FGD3

#### Workplace hierarchy

Within the healthcare facility hierarchy, MMs appeared to be the lowest in the pecking order, and as such had little available infrastructure to work with, for example, dedicated workspaces and/or counseling rooms.

We don’t have an office, no place to sit. When you come to work, anywhere you see, you just drop your bag there and hang around.—Intervention MM FGD3

The lack of working space presented a privacy challenge when MMs counseled their clients.

We are supposed to have something like a counseling room because we have issues with privacy. When you want to counsel women you will start running up and down, looking for where you and the client will sit that will be convenient and you won’t have people listening in and all that.—Intervention MM FGD3

Sometimes, if you want to talk to the women, you may find it difficult to get a conducive place to talk to them in the hospital and this issue [HIV] is not something you talk about in the open to the hearing of others.—Control MM FGD1

In some instances, even the lowest-cadre clinic staff wielded illegitimate authority over MMs, further distracting them from their primary duties:

…The attendants are ordering us about, telling us we have not done this or that.—Control Group FGD 3

#### Recruitment process and legitimacy

HCW actions may be unsupportive of MMs’ work partly because they may not consider MMs legitimate, trained healthcare workers:

We don’t have ID cards, no uniforms....—Intervention MM FGD3

While HCWs undergo a rigorous recruitment process that requires academic qualifications and licenses, the MM recruitment and engagement process lies outside the formal health sector, often supported by foreign donor-funded (in other words, non-Nigerian) grants. This seems to further delegitimize and alienate MMs with respect to their status among formal health workers.

[HCWs] said we don’t work with a certificate and are not members of staff. Even if we are staff, we are not learnѐd.—Intervention MM FGD3

#### Stigma and discrimination from healthcare workers

In more extreme cases, HCWs discriminated against MMs on the basis of their HIV-positive status and this affected MMs’ work.

The former in-charge didn’t even allow us to come close to his office; he sent us away as soon as we got close because we are HIV-positive. But the new one we have now doesn’t discriminate.—Intervention MM FGD1

Sometimes, they [HCWs] look down on us and embarrass us all the time…They hinder me from doing some things I am supposed to do in the facility. We find it so difficult to work with them.—Control MM FGD3

Some of the nurses at my site stigmatize in the way they treat us. They treat us as if it is because of being wayward that we have HIV.—Intervention MM FGD1

Some HCWs’ actions seemed to be fuelled by fear that MMs would infect them with HIV:

Some of them are like that at my facility. They think that maybe as you are interacting with them or working you will transmit the virus to them.—Control MM FGD1

#### Financial remuneration for mentor mothers

MMs confirmed that their stipends were critically needed as part (or all) of their livelihood:

Without these stipends, some of us will not survive because we have no other source of income.—Control MM FGD3

It helps pay my children’s school fees. I have three children; my husband pays for two and I pay for one.—Intervention MM FGD2

Unfortunately, in a handful of cases, some MMs faced demands for part of their stipends to be paid to supervising HCWs.

Our salary that they give us…our clinic in-charge collects 3000 naira [~$8.50] and says we must give it to her whether we like it or not …that means we are being forced. So we agreed; every month we give her 3000 naira each. It got to a point that when we don’t give her the money, we get into trouble with her.—Control MM FGD3

#### Mentor mother productivity and work performance

In this study setting, the manner in which HCWs relate to MMs has significant consequences for the latter’s work performance and job satisfaction.

In instances where MMs were made to perform extraneous tasks, it was often to the detriment of their clients/primary tasks.

The facility staff sometimes ask us to sweep and mop the facility. Even when our clients are around, they will insist that we must finish the sweeping and mopping before we attend to them.*—*Intervention MM FGD4

Unrelated tasks sometimes deny me from carrying out my mentor mother responsibilities. They [HCWs] will always tell us to leave our mentor mother roles and fill in for them.*—*Intervention MM FGD1

Every day we have to go to the facility. If we do not go it becomes a problem. So we do not get time to visit our clients. Sometimes even on Saturdays and Sundays, we are in the facility. We should know the number of days we are to go to the facility so that we can have client home visit days.—Control MM FGD3

#### Mentor mother job satisfaction and opportunities for formal integration

Despite the aforementioned challenges, there was a clearly emerging theme of MMs’ devotion to and enjoyment of their work, partly due to income but largely because the opportunity to support other women living with HIV to deliver HIV-free infants.

We thank God we are being paid, it is better than nothing at all and we enjoy the work. We like the outcomes we get after the job is done because the infants of our clients are negative.—Control MM FGD1

The most rewarding thing in this job is that we are impacting lives. The joy of the lives you impact drives you. You become a model in the community. This morning a little girl ran towards me and I could not recognize her but she reminded me of how I helped her mother. All these kinds of things encourage you, but the money is also important.—Intervention MM FGD1

Job satisfaction notwithstanding, MM expressed a desire for their workforce to be formally integrated with opportunities for advancement in the healthcare system. They understood the importance of integration, because MMs are currently neither state nor federal workers; as such, stipends are paid by donor-funded projects that are time-bound. Health facilities often do not have the financial means to sustain MM activities when donor projects end. Therefore, being absorbed as routine facility staff will give MMs more stable employment and much-needed income in an environment characterized by high levels of unemployment.

If you can move us forward, why not? Put us in the clinic as attendants.*—*Control MM FGD3

In my view when we work for long, the hospital should retain us as staff since we can read and write and perform other duties.*—*Control MM FGD1

I do blood pressure checks, new registrations for the women, I assist the clinic staff. Some of the mentor mothers have spent up to six years on the job doing mentoring. I think they should consider people like that [for higher-level jobs and pay].*—*Intervention MM FGD3

## Discussion

Our study provides detailed insight into the working conditions of mentor mothers and their professional relationships with healthcare workers in North-Central Nigeria. We have highlighted a mixture of positive and negative examples that on one hand demonstrate supportive working environments in some instances; however, there were also other instances where MMs’ working environments were less than conducive, largely due to tenuous relationships with HCWs. Some of the issues emerging were stigma and discrimination on the basis of MMs’ HIV-positive and non-formal work status, unclear scope of work at the facility level, and assignment of non-relevant tasks by HCWs.

Our findings support those of prior studies that have reported issues with lack of recognition, complementarity, and integration of lay HIV health worker roles vis-à-vis HCW roles in sub-Saharan Africa [4, 10, 11, 13]. The non-integrated, poorly structured programs in which MMs and similar lay health workers operate may actually limit their impact in the roles for which they were engaged. In our study, MMs discussed being alienated by HCWs on the basis of their non-formal work status. For example, the lack of training certificates and identity cards made MM vulnerable to dismissive treatment by some HCWs. Given the continued threat of vertical transmission to the HIV/AIDS elimination agenda [22], the opportunity to capitalize on the gains from maternal peer support in PMTCT cannot be taken for granted.

A poorly defined scope of work—especially at the facility level—was a major complaint from MMs in our study. Vagueness in job descriptions for lay health workers in HIV and PMTCT have been reported among MMs as well as other lay health workers in HIV programs [4, 11, 14, 16]. The non-formal work status of MMs and other lay health workers in HIV may contribute to the persistence of poorly defined scopes of work. This may also perpetuate HCWs’ practice of assigning non-relevant tasks to MMs, which distract them from core duties. Similar experiences have been reported among HIV peer educators in Ghana [12] and expert mothers in Malawi and Zimbabwe [4]. However, as demonstrated by the MoMent study, providing structure can significantly improve the impact of lay peer support on maternal-infant outcomes in PMTCT [24, 25]. However, MM scope of work at both facility and community-level must be well-defined, along with oriented input and buy-in from both experienced and new HCWs. The introduction of structure and standards can improve the quality and sustainability of peer support while harnessing the unique motivation of people living with HIV.

In addition to their non-formal work status, MMs’ HIV-positive status also factored into some HCWs’ attitudes towards to them. MMs reported experiencing HIV-related stigma and discrimination from the HCWs they were working with. Stigma and discrimination from HCWs towards clients is well-documented [39–42], but our study additionally highlights that directed from HCWs towards MMs. Health systems that engage MMs and other people living with HIV should be aware of this and make provisions for HCW sensitization and advocacy/protections against workplace stigma and discrimination.

In our study, payment for services was noted to be critical to MMs’ motivation to work. While stipends were provided to all MoMent study MMs for the purpose of client home visits and phone calls [4, 35], these funds were also used for MMs’ livelihood. Both paid and unpaid models of peer support have been implemented in HIV programs, all having different degrees of impact [4, 11]; however, head-to-head comparisons of paid and unpaid peer support models within the same study setting are lacking. In their analysis of lay HIV health worker programs in sub-Saharan Africa, Herman et al. report that adequate remuneration in the setting of quality supervision and continuous training is critical for quality and sustainability [10]. Cataldo et al. report similar findings from their synthesis of expert mother studies (including MoMent) in Malawi, Nigeria, and Zimbabwe, noting that adequate remuneration and training are likely to maximize the impact of these interventions in PMTCT [4]. That said, instances, however rare, of MM stipend “garnishing” by HCWs are unacceptable and unethical, and avenues for reporting and addressing these phenomena need to be available to MMs. During the MoMent study, the opportunity for this type of corruption to occur was minimized by paying all MM through their bank accounts and not by cash (via HCWs/clinic administrators), creating a safe avenue for all MMs to report such cases with minimal retaliation, and within the routine PMTCT program, involving local chapters of the Network of People Living with HIV/AIDS in Nigeria in the MM program structure. These local chapters also serve as potential pathways for MMs to report issues at work that may be taken up to funding implementing partners and/or the local health authorities.

While we have discussed MM-HCW tensions in the workplace, it is noteworthy that MMs are engaged to complement and not supplant HCWs’ jobs. As other studies have reported, much of the tensions between HCWs and lay health workers stem from poorly defined lay worker roles and HCWs’ fear of their roles being usurped from “encroachment of territory” [10, 12–14]. Thus, training and empowering MMs may work against them in their relationships with HCWs at the facility level. It is interesting to note that in our study, MMs mentioned little of tensions with HCWs or poorly defined scopes of work with regard to community-level MM activities. We suggest positioning MMs as clearly defined task shifting resources at the facility level, while protecting time for MMs to perform their community-level duties. Data on the costs and cost-effectiveness of PMTCT peer support programs have been encouraging [43] and further support the call for their standardization, integration, and scale-up.

The potential impact of MoMent’s MM program structure on MM working conditions should also be mentioned. Part of the intervention package included supportive supervision from knowledgeable, PMTCT-trained staff who acted as advocates for their assigned MMs. While not reported here, intervention arm MMs have noted how collegiate support from their study-assigned MM supervisors made them feel valued and helped them cope with job-related stress [4]. The supportive element of the supervision may very well have contributed to better MM client outcomes in the prospective MoMent study by way of higher-quality, more impactful MM counseling [24].

Our study is limited in that only the views and experiences of MMs are presented here. Our approach to the MM FGDs was to gather information on their experiences and working conditions during the MoMent study. While we interviewed HCWs (among many stakeholders) to assess acceptability of MMs as part of the formative aspect of MoMent [35, 44], we did not interview HCWs for the latter FGDs—they were limited to MMs only. Obtaining HCWs’ views on the issues explored here may have yielded additional perspectives on MM working conditions and roles. Additionally, exploration of community-level challenges faced by MMs can potentially fill in prevailing gaps in understanding their working conditions; this was not addressed in this paper. Lastly, our study was not designed to explore HCW-related experiences of MMs with shorter versus longer-term engagements; it is thus difficult to tell whether HCW-MM relationships have changed over time, for instance before MoMent and during/after MoMent. However, other reports published before this paper point to similar prevailing conditions for lay workers in HIV, albeit not in Nigeria. It appears not much has changed, likely because not much attention has been paid to developing and implementing solutions.

## Conclusions

Mentor mothers are functioning as peer counselors, community health workers, and task shifting resources and can potentially serve as mental health and domestic violence resources [45]. In HIV programs, there is a unique advantage in engaging people living with HIV to deliver HIV-related services to their peers. MMs are critical to the success of PMTCT programs in high-burden, low-income countries like Nigeria. Findings from impact evaluation studies such as MoMent provide the impetus to make accommodations for MMs within the formal health sector. This involves formally adopting and integrating structured MM programs nationwide, and sustaining them through country- and/or state-level domestic funding rather than the current (and dwindling) donor support. To capitalize on their motivation and to maximize their impact, MMs need to occupy a well-defined and well-supported niche that is minimally threatening to formal healthcare workers—so that MMs can be seen as “one of them.”

## Abbreviations

FGD: Focus group discussion
HCW: Healthcare worker
MM: Mentor mother
PMTCT: Prevention of mother-to-child transmission of HIV
WHO: World Health Organization

## References

[1] Baek C, Mathambo V, Mkhize S, Friedman I, Apicella L, Rutenberg N (2007). Key findings from an evaluation of the mothers2mothers program in KwaZulu-Natal, South Africa, Horizons Final Report.

[2] Futterman D, Shea J, Besser M, Stafford S, Desmond K, Comulada W (2010). Mamekhaya: a pilot study combining a cognitive-behavioral intervention and mentor mothers with PMTCT services in South Africa. AIDS Care.

[3] McColl K (2012). Mentor mothers to prevent mother-to-child transmission of HIV. BMJ.

[4] Cataldo F, Sam-Agudu NA, Phiri S, Shumba B, Cornelius LJ, Foster G (2017). The roles of expert mothers engaged in prevention of mother-to-child transmission (PMTCT) programs: a commentary on the INSPIRE studies in Malawi, Nigeria, and Zimbabwe. J Acquir Immune Defic Syndr.

[5] Rotheram-Borus M, Richter L, Van-Rooyen H, van-Heerden A, Tomlinson M, Stein A (2011). Project Masihambisane: a cluster randomised controlled trial with peer mentors to improve outcomes for pregnant mothers living with HIV. Trials.

[6] Schneider H, Lehmann U (2010). Lay health workers and HIV programmes: implications for health systems. AIDS Care.

[7] Flynn DE, Johnson C, Sands A, Wong V, Figueroa C, Baggaley R (2017). Can trained lay providers perform HIV testing services? A review of national HIV testing policies. BMC Res Notes.

[8] Lewin S, Munabi-Babigumira S, Glenton C, Daniels K, Bosch-Capblanch X, van Wyk BE, et al. Lay health workers in primary and community health care for maternal and child health and the management of infectious diseases. Cochrane Database Syst Rev. 2010;(3):Cd004015. 10.1002/14651858.CD004015.pub3. 10.1002/14651858.CD004015.pub3 PMC6485809 20238326

[9] Glenton C, Colvin CJ, Carlsen B, Swartz A, Lewin S, Noyes J, et al. Barriers and facilitators to the implementation of lay health worker programmes to improve access to maternal and child health: qualitative evidence synthesis. Cochrane Database Syst Rev. 2013;(10):Cd010414. 10.1002/14651858.CD010414.pub2. 10.1002/14651858.CD010414.pub2 PMC6396344 24101553

[10] Hermann K, Van Damme W, Pariyo GW, Schouten E, Assefa Y, Cirera A (2009). Community health workers for ART in sub-Saharan Africa: learning from experience--capitalizing on new opportunities. Hum Resour Health.

[11] Mwai GW, Mburu G, Torpey K, Frost P, Ford N, Seeley J (2013). Role and outcomes of community health workers in HIV care in sub-Saharan Africa: a systematic review. J Int AIDS Soc.

[12] Dapaah J, Moyer E (2013). Dilemmas of patient expertise: people living with HIV as peer educators in a Ghanaian hospital. Ghana Stud.

[13] Schneider H, Hlophe H, van Rensburg D (2008). Community health workers and the response to HIV/AIDS in South Africa: tensions and prospects. Health Policy Plan.

[14] Yakam JC, Gruenais ME (2009). Involving new actors to achieve ART scaling-up: difficulties in an HIV/AIDS counselling and testing centre in Cameroon. Int Nurs Rev.

[15] Dlamini-Simelane T, Moyer E (2017). Task shifting or shifting care practices? The impact of task shifting on patients’ experiences and health care arrangements in Swaziland. BMC Health Serv Res.

[16] Cataldo F, Kielmann K, Kielmann T, Mburu G, Musheke M (2015). ‘Deep down in their heart, they wish they could be given some incentives’: a qualitative study on the changing roles and relations of care among home-based caregivers in Zambia. BMC Health Serv Res.

[17] World Health Organization (2008). Task shifting : rational redistribution of tasks among health workforce teams : global recommendations and guidelines.

[18] Iwu EN, Holzemer WL (2014). Task shifting of HIV management from doctors to nurses in Africa: clinical outcomes and evidence on nurse self-efficacy and job satisfaction. AIDS Care.

[19] Kredo T, Adeniyi FB, Bateganya M, Pienaar ED. Task shifting from doctors to non-doctors for initiation and maintenance of antiretroviral therapy. Cochrane Database Syst Rev. 2014;(7):Cd007331. 10.1002/14651858.CD007331.pub3. 10.1002/14651858.CD007331.pub3 PMC11214583 24980859

[20] Callaghan M, Ford N, Schneider H (2010). A systematic review of task-shifting for HIV treatment and care in Africa. Hum Resour Health.

[21] UNAIDS (2017). HIV estimates with uncertainty bounds 1990-2016.

[22] UNAIDS (2016). On the fast track to an AIDS Free Generation: the incredible journey of the global plan towards the elimination of new hiv infections among children by 2015 and keeping their mothers alive.

[23] Nigeria National Agency for the Control of AIDS (2016). Fact sheet: prevention of mother-to-child transmission of HIV (PMTCT), Nigeria, 2016.

[24] Sam-Agudu NA, Ramadhani HO, Isah C, Anaba U, Erekaha S, Fan-Osuala C (2017). The impact of structured mentor mother programs on 6-month postpartum retention and viral suppression among HIV-positive women in rural Nigeria: a prospective paired cohort study. J Acquir Immune Defic Syndr.

[25] Sam-Agudu NA, Ramadhani HO, Isah C, Erekaha S, Fan-Osuala C, Anaba U (2017). The impact of structured mentor mother programs on presentation for early infant diagnosis testing in rural North-Central Nigeria: a prospective paired cohort study. J Acquir Immune Defic Syndr.

[26] Rotheram-Borus MJ, Richter LM, van Heerden A, van Rooyen H, Tomlinson M, Harwood JM (2014). A cluster randomized controlled trial evaluating the efficacy of peer mentors to support South African women living with HIV and their infants. PLoS One.

[27] Shroufi A, Mafara E, Saint-Sauveur JF, Taziwa F, Vinoles MC (2013). Mother to mother (M2M) peer support for women in prevention of mother to child transmission (PMTCT) programmes: a qualitative study. PLoS One.

[28] Richter L, Rotheram-Borus MJ, Van Heerden A, Stein A, Tomlinson M, Harwood JM (2014). Pregnant women living with HIV (WLH) supported at clinics by peer WLH: a cluster randomized controlled trial. AIDS Behav.

[29] The Ethiopia Network for HIV/AIDS Treatment Care and Support Program (2014). The role of mother mentors in supporting HIV-positive mothers.

[30] Phiri S, Tweya H, van Lettow M, Rosenberg NE, Trapence C, Kapito-Tembo A (2017). Impact of facility- and community-based peer support models on maternal uptake and retention in Malawi's option B+ HIV prevention of mother-to-child transmission program: a 3-arm cluster randomized controlled trial (PURE Malawi). J Acquir Immune Defic Syndr.

[31] Blattner W, Dakum P, Osotimehin B, Nasidi A, Abimiku A, Celentano D, Beyrer C (2009). Public health aspects of HIV/AIDS – Nigeria and West Africa. Public health aspects of HIV/AIDS in low and middle income countries.

[32] Federal Ministry of Health Nigeria (2014). Task shifting/task-sharing policy for essential health care services in Nigeria.

[33] Kyakuwa M, Hardon A, Goldstein Z (2012). “The adopted children of ART”: expert clients and role tensions in ART provision in Uganda. Med Anthropol.

[34] Zachariah R, Ford N, Philips M, Lynch S, Massaquoi M, Janssens V (2009). Task shifting in HIV/AIDS: opportunities, challenges and proposed actions for sub-Saharan Africa. Trans R Soc Trop Med Hyg.

[35] Sam-Agudu NA, Cornelius LJ, Okundaye JN, Adeyemi OA, Isah HO, Wiwa OM (2014). The impact of mentor mother programs on PMTCT service uptake and retention-in-care at primary health care facilities in Nigeria: a prospective cohort study (MoMent Nigeria). J Acquir Immune Defic Syndr.

[36] Manji-Obadiah G, Saunders E, Fan-Osuala C, Nta I, Sam-Agudu N (2017). Client evaluation of peer counselor performance in a rural PMTCT program in Nigeria.

[37] Birt L, Scott S, Cavers D, Campbell C, Walter F. Member checking: a tool to enhance trustworthiness or merely a nod to validation? Qual Health Res. 2016;26(13):1802–11. 10.1177/1049732316654870 27340178

[38] Glaser B, Strauss A. The discovery of grounded theory: strategies for qualitative research. Chicago: Aldine Transaction Publishers; 2009.

[39] Feyissa GT, Abebe L, Girma E, Woldie M (2012). Stigma and discrimination against people living with HIV by healthcare providers, Southwest Ethiopia. BMC Public Health.

[40] Vorasane S, Jimba M, Kikuchi K, Yasuoka J, Nanishi K, Durham J (2017). An investigation of stigmatizing attitudes towards people living with HIV/AIDS by doctors and nurses in Vientiane, Lao PDR. BMC Health Serv Res.

[41] Reis C, Heisler M, Amowitz LL, Moreland RS, Mafeni JO, Anyamele C (2005). Discriminatory attitudes and practices by health workers toward patients with HIV/AIDS in Nigeria. PLoS Med.

[42] Dako-Gyeke M, Dako-Gyeke P, Asampong E (2015). Experiences of stigmatization and discrimination in accessing health services: voices of persons living with HIV in Ghana. Soc Work Health Care.

[43] Wynn A, Rotheram-Borus MJ, Leibowitz AA, Weichle T, Roux IL, Tomlinson M (2017). Mentor mothers program improved child health outcomes at a relatively low cost in South Africa. Health Aff (Millwood).

[44] Sam-Agudu N, Cornelius LJ, Okundaye JN, Adeyemi OA, Isah C, Isah HO (2014). The MoMent study: acceptability of mentor mothers as a PMTCT intervention in rural North-Central Nigeria.

[45] Schneider H, Okello D, Lehmann U (2016). The global pendulum swing towards community health workers in low- and middle-income countries: a scoping review of trends, geographical distribution and programmatic orientations, 2005 to 2014. Hum Resour Health.

